# Population growth of *Varroa destructor* (Acari: Varroidae) in honey bee colonies is affected by the number of foragers with mites

**DOI:** 10.1007/s10493-016-0022-9

**Published:** 2016-02-24

**Authors:** Gloria DeGrandi-Hoffman, Fabiana Ahumada, Victor Zazueta, Mona Chambers, Geoffrey Hidalgo, Emily Watkins deJong

**Affiliations:** Carl Hayden Bee Research Center, USDA-ARS, 2000 East Allen Road, Tucson, AZ USA; AgScience Consulting LLC, Tucson, AZ USA

**Keywords:** Migration, Population models, Population dynamics, Parasite dispersal

## Abstract

Varroa mites are a serious pest of honey bees and the leading cause of colony losses. Varroa have relatively low reproductive rates, so populations should not increase rapidly, but often they do. Other factors might contribute to the growth of varroa populations including mite migration into colonies on foragers from other hives. We measured the proportion of foragers carrying mites on their bodies while entering and leaving hives, and determined its relationship to the growth of varroa populations in those hives at two apiary sites. We also compared the estimates of mite population growth with predictions from a varroa population dynamics model that generates estimates of mite population growth based on mite reproduction. Samples of capped brood and adult bees indicated that the proportion of brood cells infested with mites and adult bees with phoretic mites was low through the summer but increased sharply in the fall especially at site 1. The frequency of capturing foragers with mites on their bodies while entering or leaving hives also increased in the fall. The growth of varroa populations at both sites was not significantly related to our colony estimates of successful mite reproduction, but instead to the total number of foragers with mites (entering and leaving the colony). There were more foragers with mites at site 1 than site 2, and mite populations at site 1 were larger especially in the fall. The model accurately estimated phoretic mite populations and infested brood cells until November when predictions were much lower than those measured in colonies. The rapid growth of mite populations particularly in the fall being a product of mite migration rather than mite reproduction only is discussed.

## Introduction

The parasitic varroa mite, *Varroa destructor* Anderson & Trueman, is a serious pest to honey bees and a major cause of colony losses worldwide (Genersch et al. 2010; Guzman-Novoa et al. 2010; van Dooremalen et al. 2012). Varroa can devastate colonies that are highly infested due to effects from parasitism and transmission of viruses. However, if colonies are established with low varroa populations, it should take more than a year before they become highly infested even if untreated (DeGrandi-Hoffman and Curry 2004; Genersch 2010). Varroa reproductive rates are relatively low (Fries et al. 1994; Martin 1995a, b, 1998; de Guzman et al. 2008) even when considering that mated female mites can have 2–3 reproductive cycles (Fries and Rosenkranz 1996; Martin and Kemp 1997) and reproduce 1.3–1.5 mated daughter mites in each cycle (Martin 1994;1995). Often though, mite populations in the late fall can be unexpectedly large even if miticides are applied in late summer (Le Conte et al. 2010; DeGrandi-Hoffman et al. 2014). This suggests that factors other than reproduction might be contributing to the growth of the mite population.

One explanation for the rapid increase in mite numbers especially in the fall is that mites move among colonies by attaching to foragers. Mites could attach to foragers when they rob weak colonies collapsing from high mite infestations (Sakofski et al. 1990; Frey et al. 2011). Drifting foragers carrying mites also could contribute to the movement of varroa among colonies. The rate of mite migration depends on the number of colonies in surrounding areas (up to 1.5 km) and levels of mite infestation (Sakofski et al. 1990; Greatti et al. 1992; Goodwin et al. 2006; Frey et al. 2011).

In a previous study, we measured mite populations from spring through fall to determine the effects of different miticide treatment schedules (DeGrandi-Hoffman et al. 2014). Miticides were applied when the colonies were established from packages in the spring, and initial mite populations were low. We measured colony and mite populations until the fall and compared them with predicted population sizes generated from a model of honey bee colony and varroa population growth (DeGrandi-Hoffman and Curry 2004). The predictions were based on colony size, number of miticide treatments and mite reproduction. Predicted mite population growth was similar to the actual measurements until the fall when the actual mite populations increased sharply. The rapid growth of the mite populations could not be accounted for in the model by mite reproduction alone. We concluded that the increase in mite numbers could be due to other factors including mite migration.

Most migration of varroa into colonies occurs in the late summer and fall (Sakofski et al. 1990; Frey et al. 2011). This was determined by measuring mite drop on sticky boards in colonies established with few or no mites (Sakofski et al. 1990; Kraus and Page 1995; Frey et al. 2011; Frey and Rosenkrantz 2014). The mites on the sticky boards were assumed to have entered the hive on foragers from other colonies. When mites attach to foragers, they interfere with the bees’ homing ability and this could cause foragers to drift to other colonies and spread varroa (Kralj and Fuchs 2006).

In this study, we monitored varroa populations in colonies while determining the proportion of foragers entering and leaving hives with mites on their bodies. If the proportion of foragers with mites (FWM) substantially contributes to the mite population in colonies, there should be significant correlations between FWM and both the proportion of infested brood cells and adult bees with phoretic mites. Furthermore, if actual estimates of infested brood cells and adult bees with phoretic mites are significantly higher than predictions generated by the honey bee colony-varroa population dynamics model this would suggest that FWM are entering hives from other colonies and contributing to the unexpected increases in mite populations.

## Materials and methods

Colonies were established from package bees in April of 2014 at two locations: the University of Arizona West Agriculture Facility, Tucson, AZ, USA (site 1) and the Red Rock Agricultural Facility, Red Rock, AZ, USA (site 2). Twenty hives were established at site 1 and 21 hives at site 2. The sites were 48 km apart. These areas are in mid-altitude desert climates (latitude and longitude: N32°13′18″, W110°55′32″) where temperatures are conducive for honey bee flight until November (average November min–max: 8 and 23 °C). All colonies initially contained approximately 9000 adult bees and a laying queen. There were managed colonies in nearby apiaries at both sites. Varroa were controlled in nearby apiaries with various commercial miticide treatments to prevent colony loss.

### Estimating colony populations

Frames of bees and brood were measured monthly in all hives at both sites from May until November using methods described in DeGrandi-Hoffman et al. (2008, 2014). Areas on frames with brood and bees were estimated on both sides of each frame using a grid with 5 × 5 cm squares that covered the entire side of a comb. The grid was placed above each side of a comb and the number of squares with bees or brood was estimated. Estimates of areas covered by bees or brood was summed for all frames in the colony and used to estimate the size of the adult bee and brood population.

### Estimating varroa population density in colonies

Initial populations of phoretic mites were estimated in all hives 5 days after the colonies were established and then monthly afterwards until November. The initial estimate occurred before there was sealed brood in the colonies. Phoretic varroa populations were estimated using the ‘sugar shake’ method. We brushed approximately 300 bees into glass jars with wire screen lids. Powdered sugar (0.5–1.0 tablespoons) was added to each jar through the wire screen. The jar was rolled gently to cover the bees and then set aside for 2–3 min. The jars were inverted and shaken vigorously over a white aluminum pan containing 2.5 cm of water until there was no sugar left in the jars. The mites were counted in the pan, and the bees were placed back in each colony. The mite counts were converted to ‘mites per 100 bees’ (DeGrandi-Hoffman et al. 2014). The total phoretic mite population in the colony was estimated by: mites per 100 bees × frames covered with adult bees × 2506 bees per frame (DeGrandi-Hoffman et al. 2014).

The proportion of worker cells infested with mites and mite reproduction rates were estimated monthly beginning in May and ending in November. There were not enough drone cells in the colonies to generate meaningful data, so these were not sampled. We uncapped cells containing purple-eyed tan pupae within 24 h of emergence on all brood frames in the colony (80–100 cells per colony), and recorded the proportion with a foundress mite (i.e., fully pigmented adult female). These were classified as ‘infested cells’. We recorded the number of infested cells that had a male mite and adult females that were fully developed but not completely pigmented to estimate the proportion of infested cells with successful reproduction. Cells containing deutonymphs or protonymphs were not included because the mites would not have been mature when the bee emerged. The number of fully formed female offspring in each cell was totaled as were the number of foundress mites to estimate the number of offspring per foundress.

### Measuring the population of FWM

**Fig. 1:**
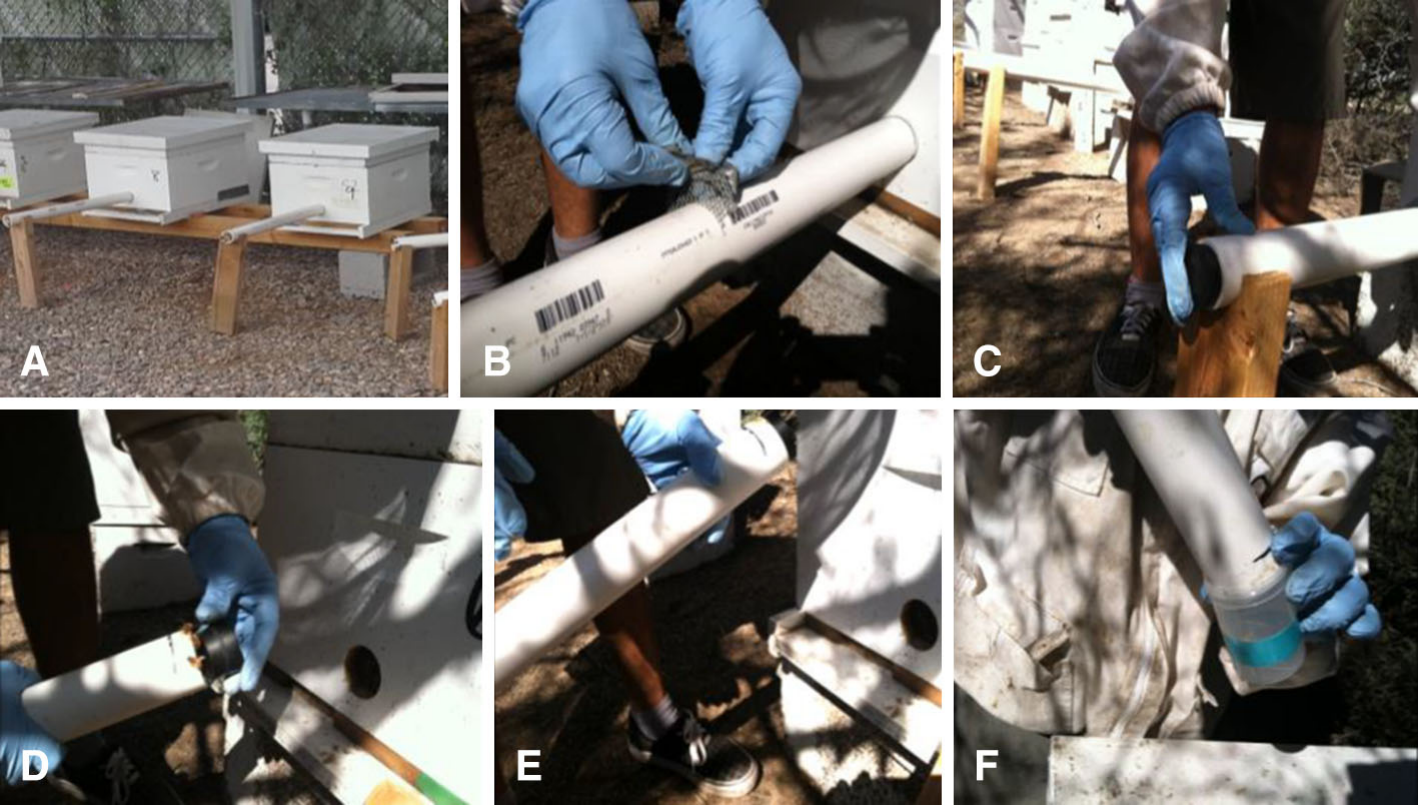
Modified hive entrance used to sample incoming and outgoing foragers. The bees entered and exited the hive using a PVC tube that was inserted into the front of the hive (**a**). The PVC tube had a slit in the *middle* where a screen could be inserted to separate foragers exiting and entering the hive (**b**). After sampling, a cork was placed in the tube facing outside to capture incoming foragers (**c**) and at the end of the tube inserted into the hive entrance (**d**) to capture outgoing foragers (**e**). The samples of incoming and outgoing foragers were shaken into jars containing 70 % ethanol to estimate the number of mites entering or leaving hives on foragers

Colony entrances were modified so that foragers exited and entered the hive through a 4.76-cm diameter PVC tube. The tube was 61 cm long. A slit was cut across the tube at the midpoint. During each sampling interval, a screen was dropped into the slit within the tube to separate foragers entering and leaving the hive (Fig. 1).

Foragers from all colonies at both sites were sampled weekly in the morning and afternoon beginning in August. We began sampling at this time because mite migration occurs with greater frequency in the late summer and fall (Sakofski et al. 1990; Kraus and Page 1995; Frey et al. 2011; Frey and Rosenkrantz 2014). Samples were taken by dropping the screen in the tube for 3 min, and then placing a rubber stopper on the end of the tube capturing incoming foragers. The tube was gently removed from the colony and a second rubber stopper was placed on the end that captured foragers leaving the colony (outgoing foragers). Samples of incoming and outgoing foragers were shaken into separate jars containing 70 % alcohol and refrigerated until the bees and mites were counted.

We determined the number of mites on incoming and outgoing foragers by adding alcohol to the collection jars and shaking the jars vigorously. The bees were then poured into a screened colander, over a white aluminum tray. Mites were counted in the tray. The bees in the colander were examined for remaining mites and then counted. Data from the samples were expressed as the proportion of incoming or outgoing FWM.

### Model simulations

Expected mite populations in colonies were generated using predictions from the honey bee colony-varroa mite population dynamics models developed by DeGrandi-Hoffman and Curry (2004). The model generates daily predictions of colony size (adults and brood), phoretic mites and infested worker and drone cells. Predictions of colony size are based on initial colony size, queen egg laying potential (i.e., the maximum number of eggs a queen can lay per day under optimum weather and colony conditions), worker longevity and weather conditions. The weather conditions used in the simulations were based on the area and time of year when the study was conducted. Predictions of mite population growth are based on initial mite population levels, colony size and growth, availability of brood of suitable age for parasitism, reproductive success rates (i.e., the proportion of infested cells where mature offspring are produced—initialized at 80 % for worker brood and 90 % for drone brood; Martin et al. 1997), and the number of daughter mites produced per mother mite (initialized as 1.5 mated daughters per foundress in worker cells and 2.6 in drone cells).

Separate sets of simulations were run for each site using average mite populations measured in May as initial conditions. Additional initial conditions for simulations were: 9000 adult bees with a randomized age distribution, a laying queen, and a starting date that was the day after packages were installed in actual colonies. Simulations were run varying worker longevity and queen egg laying potential to generate predictions of average colony sizes that were similar to actual estimates at both sites. Comparisons were made between actual and predicted phoretic mites per 100 bees and the percentage of infested cells on the dates when field sampling occurred.

### Statistical analysis

Multivariate repeated measures analyses were used to compare colony population sizes (adult bees and frames of brood), average numbers of phoretic mites per 100 bees and the percentage of infested brood cells in colonies between sites. Data were non-linear and were log_10_ transformed prior to analysis. The proportion of incoming and outgoing FWM during the study period was analysed separately for each site using multivariate repeated measures analysis to test for differences among sampling intervals (sample date and morning vs. afternoon). An additional repeated measures analysis was used to compare the populations of FWM between sites. In all cases, repeated measures analyses were run using JMP (SAS Institute, Cary, NC, USA). Multiple regressions were conducted to test for relationships between phoretic mite population growth and colony size (frames of bees and brood), proportion of infested cells, and the proportion of incoming and outgoing FWM (incoming FWM + outgoing FWM). Separate Pearson correlation analyses were used to test for relationships between FWM (incoming + outgoing) and phoretic mites and infested cells at each site. The null hypothesis in all cases was that the correlation coefficient was zero. Actual and predicted phoretic mites per 100 bees and the proportion of infested cells were compared for each sample interval using Student’s *t* tests. Regression and correlation analyses, *t* tests and all descriptive statistics were run using Minitab (State College, PA, USA). All reported means are ±SE.

## Results

### Colony growth

**Fig. 2:**
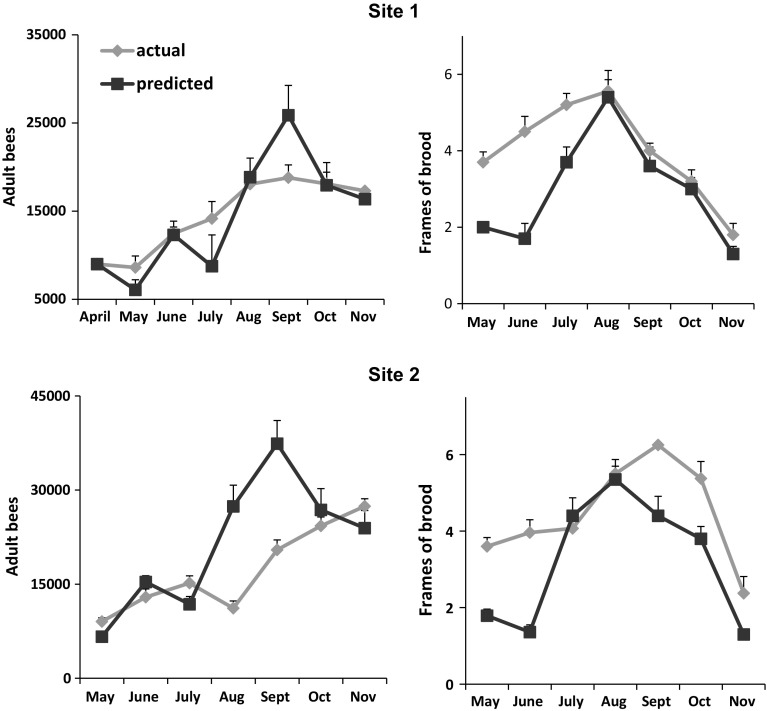
Actual and predicted growth of colony populations at 2 sites. Colonies were established from package bees (approximately 9000 bees per package) in April. Predicted values were generated from a varroa-honey bee population dynamics model initialized using actual starting colony population sizes of 9000 adult bees and no brood. Monthly averages are +SE. Adult populations did not differ between sites (F_1,24_ = 2.14, *p* = 0.16) though the interaction term time × site was significant (F_6,19_ = 21.8, *p* < 0.0001). Colonies at the two sites differed in frames of brood (F_1,24_ = 4.75, *p* = 0.04)

Colonies at site 1 grew to populations of about 19,000 ± 1377 adults (7.8 frames of bees) and 5.5 ± 0.3 frames of brood in September (Fig. 2). Populations decreased to 17,000 ± 1314 bees and 2.0 ± 0.3 frames of brood in November. At site 2, adult populations grew to about 15,000 ± 1145 bees in July, but then declined in August due to pesticide exposure. The colonies recovered and populations increased in September and peaked in November at 27000 ± 1197 adults. Brood area peaked in September at 6.2 ± 0.4 frames. Adult populations did not differ between sites (F_1,24_ = 1.97, *p* = 0.17) though the interaction term (time × site) was significant (F_6,19_ = 11.6, *p* < 0.0001). Colonies at the two sites differed in frames of brood (F_1,24_ = 6.44, *p* = 0.02). The interaction term (time × site) also was significant (F_6,19_ = 6.15, *p* = 0.001).

### Varroa populations

**Fig. 3:**
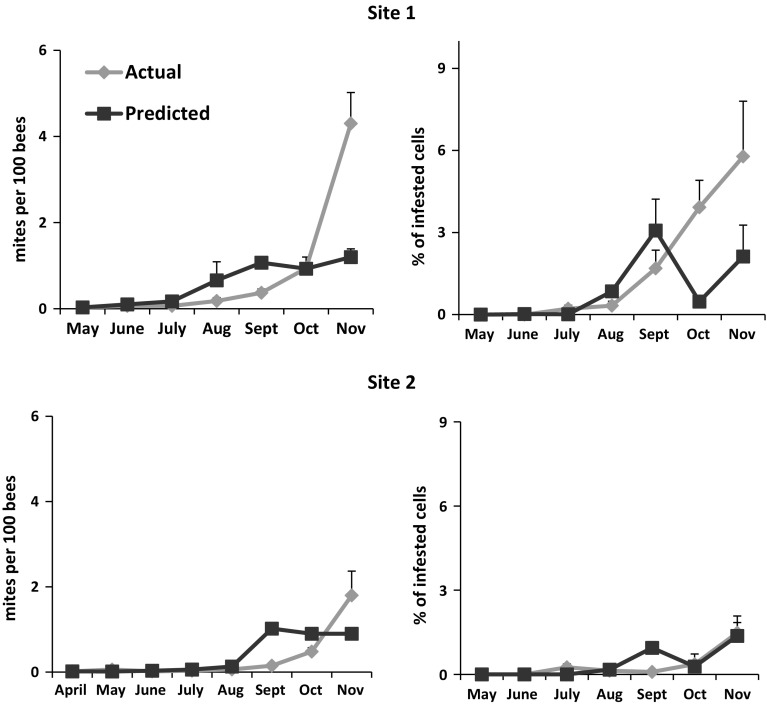
Actual and predicted monthly estimates (means + SE) of varroa mites on bees and infesting cells in honey bee colonies. Predicted values were generated from a varroa-honey bee population dynamics model initialized using actual starting colony and mite population sizes. The average number of mites per 100 bees was greater at site 1 than site 2 (F_1,24_ = 6.14, *p* = 0.021)

At site 1, we detected phoretic mites in 2 of the 20 colonies at the start of the study. In each case, the sample contained 0.33 mites per 100 bees. From June to September, mite numbers remained at <1.0 mite per 100 bees (Fig. 3). Using estimates of mites per 100 bees and frames covered with adult bees from June to September, we estimated total phoretic mite populations to be less than 100 mites. By October, colonies averaged one mite per 100 bees or 189 ± 56 phoretic mites per colony. These values were about four times higher by November when we detected 4.3 ± 0.7 mites per 100 bees or 824 ± 163 phoretic mites per colony.

Estimates of mites per 100 bees were significantly lower at site 2 compared with site 1 (F_1,24_ = 6.37, *p* = 0.019). At the start of the study, we detected varroa in four of the 21 colonies. Each of the four colonies had one mite per 100 bees. We continued to find one mite per 100 bees in less than half the colonies until October when we found mites in all but one hive. In October, colonies averaged 0.48 ± 0.09 mites per 100 bees or 128 ± 30.8 phoretic mites per colony. By November, we detected mites in all colonies with an average of 2.0 ± 0.6 mites per 100 bees or 418 ± 122 phoretic mites per colony.

Worker cells containing foundress mites were not detected in samples at either site in May or June (Fig. 3). In July and August, <1 % of the cells we sampled were infested and contained only the foundress mite. Infestation levels rose from September to November but comprised <6 % at site 1 and <2 % at site 2 of the cells we sampled. There was no significant difference in the proportion of infested worker cells between sites (F_1,18_ = 4.0, *p* = 0.06). Mites successfully reproduced a fully developed daughter mite in 16 % of the infested cells at site 1 and 8 % at site 2. We did not find any cells where the foundress produced more than a single fully developed daughter mite in the infested cell. During each sampling interval, we examined on average 1300 cells among the colonies at site 1 and 1024 at site 2.

### Proportion of FWM

At site 1, there was an average of 30–70 foragers captured either entering or leaving colonies during each sampling interval. There was no significant difference in the number of incoming and outgoing foragers during sampling intervals at either site (F_1,65_ = 0.115, *p* = 0.73) or between morning and afternoon samples (F_1,65_ = 0.397, *p* = 0.53). Thus, the likelihood of collecting FWM was not due to differences in the number of foragers we sampled.

**Fig. 4:**
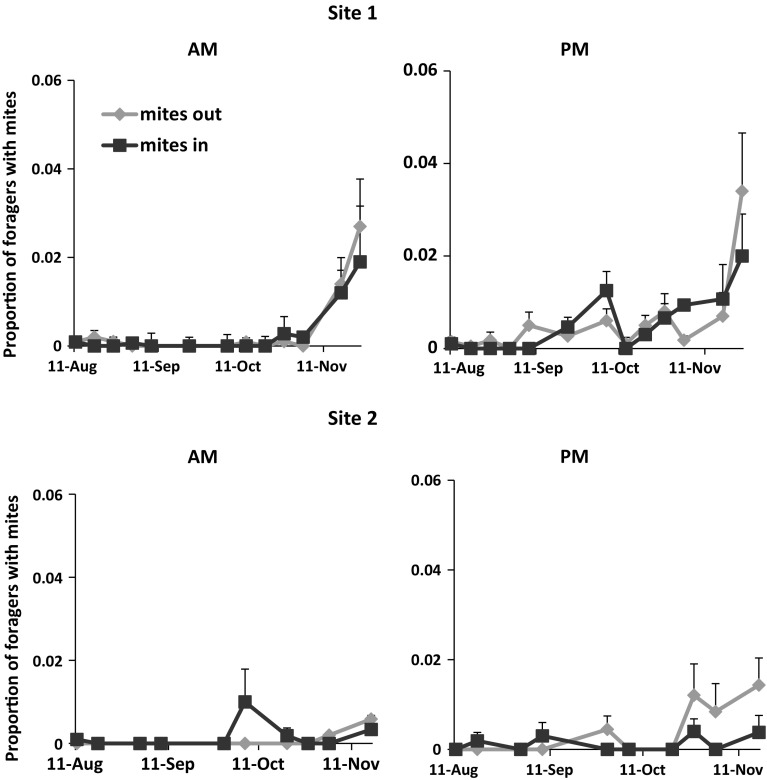
The proportion of forager bees with mites (FWM) on their bodies either entering (mites in) or leaving (mites out) hives in a 3 min interval. There were fewer FWM at site 2 than at site 1 (F_1,136_ = 4.96, *p* = 0.027)

We rarely collected FWM in August at either site (Fig. 4). In September, the frequency of FWM was increasing especially in afternoon samples. There was no significant difference in the proportion of incoming and outgoing FWM at either site (F_1,138_ = 0.03, *p* = 0.86). However, more FWM were captured in afternoon than morning samples (F_1,138_ = 9.6, *p* = 0.002). At site 1, we collected significantly more FWM in October and November than in August or September (F_10,67_ = 3.88, *p* = 0.0003). The likelihood of capturing FWM at site 2 was slightly but not significantly greater in fall months than in summer (F_9,49_ = 1.87, *p* = 0.078). Overall, there were fewer FWM at site 2 than at site 1 (F _1,136_ = 5.2, *p* = 0.023).

### Factors affecting varroa population growth

Separate analyses were conducted using data from each site to identify factors affecting mite population growth (i.e., phoretic mites and infested cells). Mites per 100 bees were significantly related to colony growth (frames of adult bees and brood) and the total incoming and outgoing FWM (Table 1). The proportion of infested cells also was related to colony growth at both sites, but not to the size of the phoretic mite population at either site (Table 2). Pearson correlation coefficients (r_p_) for FWM and phoretic mites per 100 bees were: r_p_ = 0.81, *p* < 0.0001 for site 1, and r_p_ = 0.61, *p* < 0.0001 for site 2. The proportion of cells infested with mites estimated at each sampling interval also was significantly correlated with FWM at site 1 (r_p_ = 0.03, *p* = 0.01), but not at site 2 (r_p_ = 0.155, *p* = 0.43).

**Table 1:** Multiple regression equations with factors affecting changes in phoretic varroa mite populations in colonies at two sites (site 1: R^2^ = 56 %; site 2: R^2^ = 63.6 %)

Location	Predictor	Coefficient	t	*p*
Site 1	y-Intercept	5.52	2.82	0.006
	Frames of adult bees	0.486	2.14	0.036
	Frames of brood	−1.65	4.35	<0.0001
	Infested cells (mites/100 cells)	−3.0	0.24	0.81
	Foragers with mites	87.8	5.64	<0.0001
Site 2	y-Intercept	2.6	0.77	0.45
	Frames of adult bees	0.186	0.86	0.51
	Frames of brood	3.3	1.75	0.16
	Infested cells (mites/100 cells)	0.56	0.21	0.84
	Foragers with mites	116	2.76	0.012

The ‘foragers with mites’ data were entered into the analysis as the mites on incoming and outgoing foragers

**Table 2:** Multiple regression equations with factors affecting changes in mite infestation levels in worker cells at two sites (site 1: R^2^ = 25 %; site 2: R^2^ = 45.6 %)

Location	Predictor	Coefficient	t	*p*
Site 1	y-Intercept	0.04	2.01	0.048
	Frames of adult bees	0.0058	2.60	0.012
	Frames of brood	−0.014	3.57	0.001
	Mites/100 bees	−0.0003	0.24	0.81
	Foragers with mites	0.012	0.192	0.95
Site 2	y-Intercept	0.096	0.65	0.52
	Frames of adult bees	0.225	2.23	0.038
	Frames of brood	−0.339	2.01	0.059
	Mites/100 bees	0.019	0.21	0.84
	Foragers with mites	−25.21	1.30	0.21

### Comparisons of actual and predicted varroa populations

At site 1, predicted and actual estimates of mites per 100 bees were similar until November when predicted values were significantly lower than actual (t_19_ = 4.24, *p* < 0.0001) (Fig. 3). Predictions of cell infestation percentages were similar to actual measurements until October when the predictions were significantly lower than the actual (t_17_ = 3.44, *p* = 0.003). November predictions also were lower than actual infestation levels, though the differences were not significant (t_19_ = 1.58, *p* = 0.13). During October and November, predicted and actual colony sizes (adult bees and frames of brood) did not differ significantly (Fig. 2).

Predicted mites per 100 bees at site 2 were similar to observed until November when predictions were lower than observed. However, the differences were not significant (t_10_ = 1.57, *p* = 0.15). Predictions of infested cells at site 2 were similar to observed for the entire study period. As at site 1, predicted and actual colony sizes did not differ in October and November.

## Discussion

The proportion of both adult workers with phoretic mites and infested brood cells was correlated with the frequency of FWM entering and leaving the hive. Observed and predicted mite population growth in colonies was similar when there were few FWM. However, as the frequency of collecting FWM increased in the fall, predicted mite populations (based only on mite reproduction) were much lower than observed in colonies. The combination of field data and simulations suggest that FWM were drifting in from other colonies and contributing to the growth of varroa populations in our colonies particularly in the fall.

In our study, the proportion of adult bees with phoretic mites was not related to cell infestation levels, but instead to the proportion of FWM. The levels of infested cells and successful reproduction we measured were low, and if they were underestimated, we also would underestimate the relationship between reproduction and mite population growth. Therefore, the data analysis should be interpreted cautiously. Reproductive rates might have been underestimated because we did not examine enough cells per colony. We counted mites in 80–100 cells during each sampling interval except in November when brood of suitable age was limited and we averaged about 60 cells per colony. Others recommend 200 cells per colony (Rinderer et al. 2001; de Guzman et al. 2007) to estimate infestation rates. However, these recommendations are for colonies that are larger than those we used. Our colonies were started from packages and did not have large areas of brood of suitable age for sampling 200 cells per colony especially in the first months after they were established. Low levels of reproductive success might also be related to the temperatures at the study sites. Others have reported that the growth rate of varroa populations is inversely correlated to the percentage of days with maximal temperatures >35 °C (Harris et al. 2003). Our study sites routinely had daily high temperatures between 38 and 40 °C until October. Finally, we quantified reproductive success to include only foundresses in cells containing fully formed daughter mites. Those with deutonymphs were not counted as reproductive success or offspring because the workers in the cells we sampled would have emerged within 24 h and the immature mites would not have fully developed.

To account for possible underestimation in mite reproductive from our field measurements, we ran simulations with literature values for reproductive success in worker cells (i.e., 80 % of the foundress mites invading worker cells reproduced) and numbers of offspring (1.5 fully developed daughter mites). At site 1 where FWM were consistently higher than site 2, actual estimates of the percentage of infested cells were similar to predictions until September. After this, actual infestation levels were much greater than predicted even though the predictions were based on higher rates of reproductive success. At site 2, there were fewer FWM, and estimates of infestation levels and phoretic mite populations were similar to the predicted for the entire study period. The contrast between results at the two sites might be related to differences in the frequency of capturing FWM and their contribution to mite population growth.

The proportion of FWM was similar between those entering and leaving the hive. These results differ from Kralj and Fuchs (2006) where the proportion of FWM leaving colonies was about twice as high as those returning. In that study, the foragers were marked and could be identified as originating from the colony. The samples were collected in August and September, a time when we found few FWM. Our sampling of foragers for a longer period (August–November) and in morning and afternoon revealed different trends that might lend themselves to alternative interpretations of the data. Since the number of FWM did not differ between incoming and outgoing bees, we may have been measuring a single population of FWM entering and leaving the hives. The frequency of collecting FWM seemed to increase during the day suggesting that when mites attach to foragers they remain on them until foraging ends for the day. The mites may then move to nurse bees and brood cells. This scenario, while speculative is one possible explanation for why the phoretic mite populations in the brood area and the proportion of infested cells rose, particularly at site 1, as the frequency of FWM increased.

We cannot be certain if the FWM we collected were from the same colony where the foragers originated or were from other colonies. However, if the FWM originated from the same colony we sampled and numbers of FWM entering and leaving the hive were not significantly different, we should not have seen an increase in the overall mite population or discrepancies between observed and predicted varroa populations in the fall. Furthermore, when mite populations are low as they were in our colonies (<10 % adult workers with mites), mites attach to nurse bees that they can distinguish from foragers by different cuticular chemical signatures and remain within the colony (Cervo et al. 2014). Instead, mite populations estimated from samples taken in the brood area increased at the same time as the FWM at both sites. The degree that the mite populations increased was related to the growth in the population of FWM. More FWM were detected in the fall at site 1 than at site 2, and mite populations in site 1 colonies were significantly larger.

Increases in phoretic mite populations in the fall were expected since brood rearing is declining at this time and fewer brood cells are available to infest. The model also generates an inverse relationship between phoretic mites and brood rearing in the fall. Adding to the phoretic mite population can be an influx of mites on foragers robbing colonies that are collapsing due to high mite populations (Sakofski et al. 1990; Greatti et al. 1992). We did not detect high proportions of FWM during any sampling period as would be expected if foragers were robbing heavily infested collapsing colonies. Though increases in mite numbers due to robbing probably occur, our data suggest other possible explanations for unexpected growth in mite populations. FWM comprised at most 2–3 % of all the foragers we captured in a sampling interval. These percentages are similar to those reported by Goodwin et al. (2006). The increases in the proportion of infested cells and adult bees with phoretic mites could occur from a low level entry of mites during foraging periods (especially in the fall) that over time generates mite numbers that are significantly higher than expected from reproduction alone. Similar trends have been reported by Frey and Rosenkrantz (2014), who estimated 30–60 mites invading colonies per week (or about 4–9 mites per day). Over the 3.5-month study, 266–1171 mites entered the hives; phoretic mites on adult bees increased from 0.2 to 18 % and infested cells from 0.7 to 50.8 % (Frey and Rosenkrantz 2014).

The transfer of mites to foragers is a shift in the mite behavior from attaching to nurse bees for reproduction (Kraus 1993; Kuenen and Calderone 1997) to foragers for possible dispersal. The frequency of this behavioral shift seems to increase in the fall, and might occur for several reasons. Varroa populations are at their highest levels in the fall and brood production is decreasing. There are fewer brood cells to infest so more mites are on worker bees perhaps including foragers (Sakofski et al. 1990). In hives that are highly infested with mites, the chemical profile of nurses and foragers can overlap causing mites to attach to foragers (Cervo et al. 2014). There are indications that foragers carrying varroa have low returning rates to their own colonies (Kralj and Fuchs 2006) and could be drifting to other hives. The drifting could be due to parasitism alone or infection by viruses that varroa transmit such as Deformed Wing Virus (DWV) or Israeli Acute Paralysis Virus (IAPV). Both viruses affect learning and memory (Li et al. 2013; Iqbal and Mueller 2007). DWV and IAPV titers increase with the growth of the mite population throughout the season reaching their highest levels in the fall (Francis et al. 2013). Left untreated, these colonies collapse over the winter. Viruses vectored by varroa that affect forager orientation causing them to drift could provide a mechanism for both the virus and the mite to disperse in the fall from colonies that are likely to die over the winter.

## References

[CR1] Cervo R, Bruschini C, Cappa F, Meconcelli S, Pieraccini G, Pradella D, Turillazzi S (2014). High Varroa mite abundance influences chemical profiles of worker bees and mite-host preferences. J Exp Biol.

[CR2] de Guzman LI, Rinderer TE, Frake AM (2007). Growth of *Varroa destructor* (Acari: Varroidae) populations in Russian honey bee (Hymenoptera: Apidae) colonies. Ann Entomol Soc Am.

[CR3] DeGrandi-Hoffman G, Curry R (2004). A mathematical model of Varroa mite (*Varroa**destructor* Anderson and Trueman) and honeybee (*Apis mellifera* L.) population dynamics. Int J Acarol.

[CR4] DeGrandi-Hoffman G, Wardell G, Ahumada-Secura F, Rinderer TE, Danka R, Pettis J (2008). Comparisons of pollen substitute diets for honeybees: consumption rates by colonies and effects on brood and adult populations. J Apic Res.

[CR5] DeGrandi-Hoffman G, Ahumada F, Curry R, Probasco G, Schantz L (2014). Population growth of *Varroa destructor* (Acari: Varroidae) in commercial honey bee colonies treated with beta plant acids. Exp Appl Acarol.

[CR6] deGuzman LI, Rinderer TE, Frake AM (2008). Comparative reproduction of *Varroa**destructor* in different types of Russian and Italian honey bee combs. Exp Appl Acarol.

[CR7] Francis RM, Nielsen SL, Kryger P (2013). Varroa–virus interaction in collapsing honey bee colonies. PLoS One.

[CR8] Frey E, Rosenkrantz P (2014). Autumn invasion rates of *Varroa destructor* (Mesostigmata: Varroidae) into honey bee (Hymenoptera: Apidae) colonies and the resulting increase in mite populations. J Econ Entomol.

[CR9] Frey E, Schnell H, Rosenkranz P (2011). Invasion of *Varroa destructor* into mite-free honeybee colonies under the controlled conditions of a military training area. J Apic Res.

[CR10] Fries I, Rosenkranz P (1996). Number of reproductive cycles of *Varroa jacobsoni* in honey-bee (*Apis mellifera*) colonies. Exp Appl Acarol.

[CR11] Fries I, Camazine S, Sneyd J (1994). Population dynamics of *Varroa jacobsoni*: a model and a review. Bee World.

[CR12] Genersch E (2010). Honey bee pathology: current threats to honey bees and beekeeping. Appl Microbiol Biotechnol.

[CR13] Genersch E, von Der Ohe W, Kaatz H, Schroeder A, Otten C, Buchler R, Berg S, Ritter W, Muehlen W, Gisder S (2010). The German bee monitoring project: a long term study to understand periodically high winter losses of honeybee colonies. Apidologie.

[CR14] Goodwin RM, Taylor MA, McBrydie HM, Cox HM (2006). Drift of *Varroa**destructor* infested worker honeybees to neighbouring colonies. J Apic Res.

[CR15] Greatti M, Milani N, Nazzi F (1992). Reinfestation of an acaricide-treated apiary by *Varroa jacobson*i Oud. Exp Appl Acarol.

[CR16] Guzman-Novoa E, Eccles L, Calvete Y, McGowan J, Kelly PG, Correa-Benitez A (2010). *Varroa**destructor* is the main culprit for the death and reduced populations of overwintered honey bee (*Apis**mellifera*) colonies in Ontario, Canada. Apidologie.

[CR17] Harris JW, Harbo JR, Villa JD, Danka RG (2003). Variable population growth of *Varroa destructor* (Mesostigmata: Varroidae) in colonies of honey bees (Hymenoptera: Apidae) during a 10-year period. Environ Entomol.

[CR18] Iqbal J, Mueller U (2007). Virus infection causes specific learning deficits in honeybee foragers. Proc R Soc B.

[CR19] Kralj J, Fuchs S (2006). Parasitic *Varroa destructor* mites influence flight duration and homing ability of infested *Apis mellifera* foragers. Apidologie.

[CR20] Kraus B (1993). Preferences of *Varroa jacobsoni* for honey bees *Apis mellifera* L. of different ages. J Apicult Res.

[CR21] Kraus B, Page RE (1995). Population growth of *Varroa jacobsoni* Oud. in Mediterranean climates of California. Apidologie.

[CR22] Kuenen LPS, Calderone NW (1997). Transfers of *Varroa* mites from newly emerged bees: preferences for age- and function-specific adult bees (Hymenoptera: Apidae). J Insect Behav.

[CR23] Le Conte Y, Ellis M, Ritter W (2010). Varroa mites and honeybee health: can Varroa explain part of the colony losses?. Apidologie.

[CR24] Li Z, Chen Y, Zhang S, Chen S, Li W (2013). Viral infection affects sucrose responsiveness and homing ability of forager honey bees, *Apis mellifera* L.. PLoS One.

[CR25] Martin SJ (1994). Ontogenesis of the mite *Varroa jacobsoni* Oud. in worker brood of the honeybee *Apis mellifera* L. under natural conditions. Exp Appl Acarol.

[CR26] Martin SJ (1995). Ontogenesis of the mite *Varroa jacobsoni* Oud. in drone brood of the honeybee *Apis mellifera* L. under natural conditions. Exp Appl Acarol.

[CR27] Martin SJ (1995). Reproduction of *Varroa jacobsoni* in cells of *Apis mellifera* containing one or more mother mites and the distribution of these cells. J Apicult Res.

[CR28] Martin SJ (1998). A population model for the ectoparasitic mite *Varroa* j*acobsoni* in honey bee (*Apis mellifera*) colonies. Ecol Model.

[CR29] Martin SJ, Kemp D (1997). Average number of reproductive cycles performed by *Varroa jacobsoni* in honey bee (*Apis mellifera*) colonies. J Apicult Res.

[CR30] Martin SJ, Holland K, Murray M (1997). Non-reproduction in the honeybee mite *Varroa jacobsoni*. Exp Appl Acarol.

[CR31] Rinderer TE, de Guzman LI, Delatte GT, Stelzer JA, Lancaster VA (2001). Resistance to the parasitic mite *Varroa destructor* in honey bees from far-eastern Russia. Apidologie.

[CR32] Sakofski F, Koeniger N, Fuchs S (1990). Seasonality of honeybee colony invasion by *Varroa jacobsoni* Oud. Apidologie.

[CR33] van Dooremalen C, Gerritsen L, Cornelissen B, van der Steen JJM, van Langevelde F (2012). Winter survival of individual honey bees and honey bee colonies depends on level of *Varroa destructor* infestation. PLoS One.

